# Determination of experimental domain factors of polyphenols, phenolic acids and flavonoids of lemon *(Citrus limon)* peel using two-level factorial design

**DOI:** 10.1016/j.sjbs.2021.09.022

**Published:** 2021-09-16

**Authors:** Zainol Haida, Sharin Ab Ghani, Jaafar Juju Nakasha, Mansor Hakiman

**Affiliations:** a Department of Crop Science, Faculty of Agriculture, Universiti Putra Malaysia, 43400 UPM Serdang, Selangor, Malaysia; b High Voltage Engineering Research Laboratory, Faculty of Electrical Engineering, Universiti Teknikal Malaysia, Melaka, Hang Tuah Jaya, 76100 Durian Tunggal, Melaka, Malaysia

**Keywords:** *Citrus limon*, Polyphenols, Phenolic acids, Flavonoids, Two-level factorial design

## Abstract

This study aimed to evaluate the significant extraction factors in achieving higher recovery yield of total polyphenols, phenolic acids and flavonoids content from *Citrus limon* peel using two-level factorial design. The effect of five independent factors including drying temperature (40–60 °C), methanol concentration (20–60%), extraction temperature (28–60 °C), extraction time (30–60 min) and storage duration (0–14 days) were evaluated. Among all the examined factors, results showed that drying temperature, storage duration and extraction temperature were the most significant and contributing factors affecting the total polyphenols, phenolic acids and flavonoids content of lemon peel at P < 0.05. On the contrary, methanol concentration and extraction time exhibited the least significant and contribution at P greater than 0.05. In conclusion, the experimental domain factors were successfully obtained from this experiment, Therefore, further study on optimization of the obtained factors will be conducted in the future study using response surface methodology.

## 1 Introduction

*Citrus limon* or commercially know as lemon is belongs to family Rutaceae. Lemon is one of the most popular *Citrus* species in the world after orange and mandarin (Miran et al., 2016). In other countries, lemon is known as zitrone in Germany, le citron in French, limon in Spanish and ningmeng in Chinese (Klimek-Szczykutowicz et al., 2020). Lemon is popular due to its various types of phytochemical and ample supply of vitamin C, folic acid, potassium and pectin which act as supplement to improve human health and prevent diseases (Rafiq et al., 2018, Sagar et al., 2018). Lemon is widely consumed worldwide as a fresh fruit and juice but the lemon peel is mostly discarded as a waste (Sagar et al., 2018). However, consumers are not aware that the lemon peel contains a wide range of bioactive compounds with substantial antioxidant activity which might contain useful bioactive compounds comparable to that of lemon pulps (Mahato et al., 2019). The lemon peel can be divided into epicarp or flavedo (colored peripheral surface) and mesocarp or albedo (white soft middle layer) (Rafiq et al., 2018). Since the lemon peel is considered as a waste, only several studies have been conducted on phenolics content and antioxidant activities of lemon peel. In order to maximize the phenolics content and antioxidant activities of lemon peel, optimization of extraction process is an important step need to be carried out properly.

The optimization of extraction process is a crucial step as different species or plant parts require distinct extraction procedures and conditions to yield maximum phenolic compounds and antioxidant activities due to its different characteristic and phytochemicals constituent (Azwanida et al., 2015). In the optimization of phenolics content extraction from *Citrus* species, the frequently used extraction variables are solvent concentration, liquid–solid ratio, extraction temperature and extraction time and the ranges of each variables used are varied depending on several factors including plant parts, maturity of fruits and targeted secondary metabolites (Assefa et al., 2017, Nipornram et al., 2018, Fakayode and Abobi, 2018, Colodel et al., 2018, Nishad et al., 2019, Kim and Lim, 2020).

In this current study, two-level factorial design was adopted as a statistical screening process. The two-level factorial design is a useful method for the extraction process to determine the main factor as well as interaction between the factors with minimal experimental runs. Hence, this study was conducted to screen the independent factors including drying temperature, methanol concentration, extraction temperature, extraction time and storage duration for the recovery of total polyphenols, phenolic acids and flavonoids content from lemon peel using two-level factorial design.

## 2 Materials and methods

### 2.1 Plant material

Fresh fruits of *Citrus limon* (lemon) at their mature stage were purchased from local grocery store in Selangor, Malaysia. The fruits were imported from South Africa. The fruits were cleaned thoroughly under running tap water. The fruit peel was manually separated from the flesh and wrapped with paper for drying procedure.

### 2.2 Chemicals and reagents

Gallic acid, rutin, Folin-Ciocalteu reagent, aluminium chloride, sodium carbonate and methanol were purchased from R&M chemical company and Sigma-Aldrich. All chemicals and reagents used in this study were of analytical grade.

### 2.3 Extraction process

The extraction procedure of lemon peel was carried out using maceration method. First, the dried samples which were dried at different oven temperatures (40 – 60 °C) were finely ground using a commercial blender. A total of 0.5 g of powdered sample was mixed with 25 mL of methanol at different concentrations (20 – 60%) as extraction solvent. Then, the mixtures were placed in a water bath with a temperature ranged from 28 to 60 °C and extraction time was set between 30 and 60 min. The mixtures were filtered using filter paper No. 1 and stored in the refrigerator (5 °C) for 0 to 14 days. The variables were set based on the design matrix generated by Design Expert software version 11.0 (Table 1).

**Table 1:** Parameters for the two-level factorial design.

Factor	Notation		Factor levels	
		Low (-1)	Centre point (0)	High (+1)
Drying temperature (°C)	A	40	50	60
Methanol concentration (%)	B	20	40	60
Extraction temperature (°C)	C	28	44	60
Extraction time (min)	D	30	45	60
Storage duration (day)	E	0	7	14

### 2.4 Total polyphenols content

The method described by Marinova et al. (2005) was used for total polyphenols content analysis. Briefly, the extract (50 µL) was mixed with ten-fold diluted Folin-Ciocalteu reagent (1.25 mL) and the mixture was incubated for five minutes. After the incubation, 7% sodium carbonate (1.25 mL) added and the mixture was incubated at room temperature for an hour. The change in absorbance was measured using UV–Vis spectrophotometer at 725 nm. The gallic acid was used as standard and the total polyphenols content of lemon peel was expressed as mg gallic acid equivalent per gram dry weight of sample (mg GAE/g DW).

### 2.5 Total phenolic acids content

The quantification of total phenolic acids content was conducted using method as explained by Singleton and Rossi, 1965, Haida et al., 2019. The mixture containing extract (0.5 mL), distilled water (4.5 mL) and Folin-Ciocalteu reagent (0.5 mL) were mixed and incubated for five minutes. After the incubation period, 7% sodium carbonate (5 mL) and distilled water (2 mL) were added. The reaction mixture was incubated at room temperature for 90 min and absorbance was measured at 750 nm using UV–Vis spectrophotometer. The gallic acid was used as standard and the total phenolic acids content of lemon peel was expressed as mg gallic acid equivalent per gram dry weight of sample (mg GAE/g DW).

### 2.6 Total flavonoids content

The determination of total flavonoids content from lemon peel was quantified using aluminium chloride colorimetric method as described by Marinova et al. (2005). The mixture of extract (0.5 mL), distilled water (2 mL) and 5% sodium nitrite (150 µL) were mixed thoroughly and incubated for five minutes. Then, 10% aluminium chloride (150 µL) was added to the mixture followed by 1 M sodium hydroxide (1 mL) and distilled water (1.2 mL). The reaction mixture was mixed and absorbance was measured at 510 nm using UV–Vis spectrophotometer. The rutin was used as standard and the total flavonoids content was expressed as mg rutin equivalent per gram dry weight of sample (mg RE/g DW).

### 2.7 Experimental design and statistical analysis

In this study, five extraction factorials namely drying temperature, methanol concentration, extraction temperature, extraction time and storage duration were analyzed using two-level factorial design. Each factorial including drying temperature (A, °C), methanol concentration (B, %), extraction temperature (C, °C), extraction time (D, minutes) and storage duration (days) were evaluated using two coded levels in a randomized trend with a total of 32 experimental trials (2^5^ = 32) and three replications of the centre points (Table 2). Three responses recorded were total polyphenol content (mg GAE/g DW), total phenolic acids content (mg GAE/g DW) and total flavonoids content (mg RE/g DW). The experimental runs and analysis were conducted in triplicate and the value was expressed as mean. The analysis of variance (ANOVA) was used to analyzed the p-value and significance of the model. The Design Expert 11.0 software (Stat-Ease Inc., Minneapolis, USA) was used for designing the experiment and data analysis.

**Table 2:** Design matrix and the responses of two-level factorial design.

Run	Factors					Responses		
	A	B	C	D	E	Y_1_	Y_2_	Y_3_
1	60	20	60	60	14	2.8	4.74	47.26
2	60	60	60	60	14	3.08	5.05	55.7
3	60	20	28	30	0	2.5	7.9	37.4
4	60	60	28	30	0	2.4	8.16	37.12
5	60	20	28	30	14	2.78	6.05	42.37
6	60	60	60	30	0	2.77	8.04	47.12
7	40	20	28	60	0	1.86	3.89	21.51
8	40	20	60	60	0	1.94	3.48	28.45
9	60	60	28	30	14	2.9	4.74	31.87
10	60	60	28	60	0	2.58	7.22	35.9
11	40	60	60	30	0	2.71	5.12	37.4
12	40	20	60	30	0	2.07	4.86	28.54
13	40	20	28	60	14	1.82	3.55	22.79
14	60	20	60	60	0	2.52	8.11	45.26
15	60	60	60	30	14	3.24	5.62	54.29
16	40	60	60	60	14	3.55	6.97	54.31
17	40	60	28	30	0	1.72	3.77	22.4
18	60	60	28	60	14	2.92	4.54	38.76
19	40	60	28	60	0	1.4	3.71	21.31
20	60	20	60	30	14	2.92	6.06	49.45
21	60	60	60	60	0	2.78	6.78	47.01
22	60	20	28	60	14	2.71	4.94	44.76
23^a^	50	40	44	45	7	2.58	4.52	34.04
24	60	20	28	60	0	2.45	7.42	32.34
25	40	60	60	60	0	2.62	5.24	33.62
26^a^	50	40	44	45	7	2.69	4.33	34.7
27	40	20	28	30	0	1.96	5.55	22.26
28	40	60	28	60	14	1.99	4.11	23.93
29	40	20	60	30	14	2.24	4.31	30.93
30	40	20	28	30	14	1.79	3.68	24.01
31	60	20	60	30	0	2.63	8.87	41.43
32	40	60	60	30	14	2.74	5.17	38.84
33^a^	50	40	44	45	7	2.65	4.2	34.45
34	40	20	60	60	14	2.29	5.12	34.54
35	40	60	28	30	14	2.06	4.18	22.7

A – Drying temperature (°C); B – Methanol concentration (%); C – Extraction temperature (°C); D – Extraction time (min); E – Storage duration (day); Y_1_ – Total polyphenols content (mg GAE/g DW); Y_2_ – Total phenolic acids content (mg GAE/g DW); Y_3_ – Total flavonoids content (mg RE/g DW). ^a^Centre point.

## 3 Results

### 3.1 Influence of extraction factors on the total polyphenols content

**Fig. 1:**
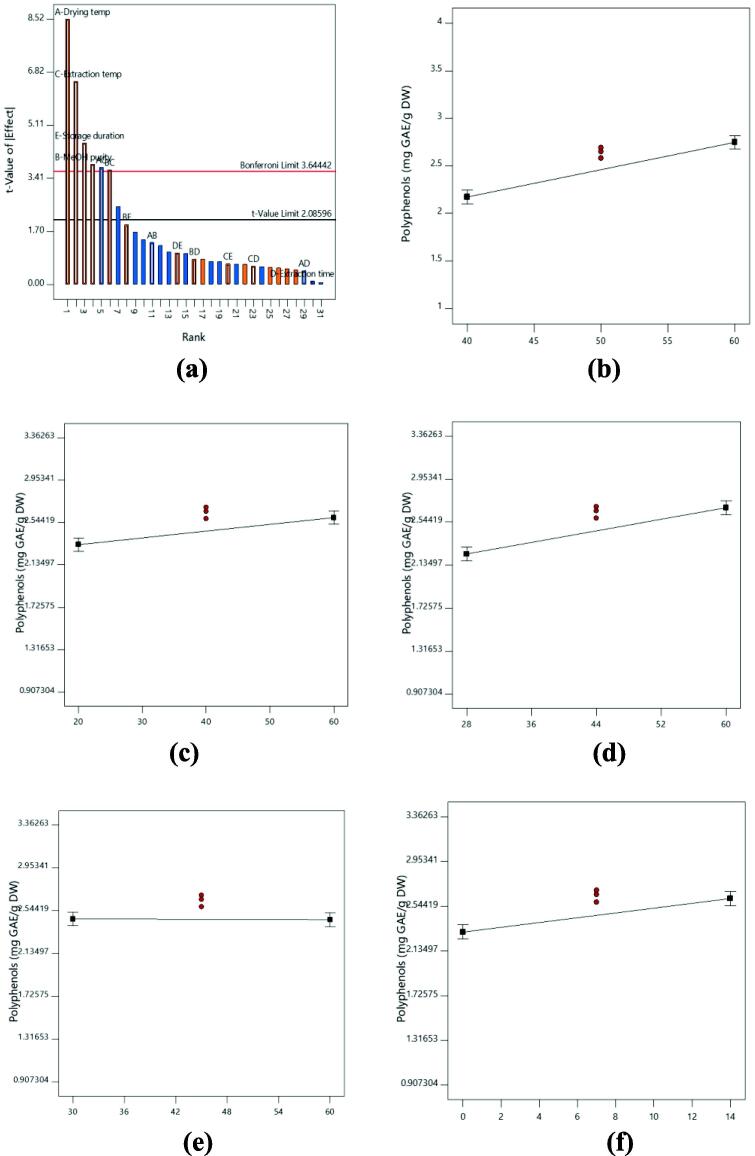
**(a)** The Pareto Chart of all factors and interactions on total polyphenols content from lemon peel; Influence of **(b)** drying temperature; **(c)** methanol concentration; **(d)** extraction temperature; **(e)** extraction time; **(f)** storage duration on the total polyphenols content recovery from lemon peel. A greater slope shows a greater influence on the recovery.

The response model was highly significant with P < 0.0001 (Table 3). Based on Fig. 1 and Table 3, it can be observed that an increase in the drying temperature, methanol concentration, extraction temperature and storage duration resulted in higher recoveries of total polyphenols content of lemon peel. In contrary, extraction time showed the least effect on total polyphenols content of lemon peel. The recovery of total polyphenols was greatly affected by drying temperature, methanol concentration, extraction temperature and storage duration with P < 0.05. Meanwhile, the extraction time was non-significant on total phenolic acids content. The effect of drying temperature was the major contributing factor with 34.91%, followed by extraction temperature (20.47%), storage duration (9.94%) and extraction temperature (7.17%), respectively (Table 4). The Pareto Chart is used to measure the sampling error of individual variable and their interaction through the standard deviation. Based on Pareto chart of total polyphenols content (Fig. 1a), the t-value limit is 2.08596. Any factor or interaction that falls below t-value will be non-significant. In Fig. 1a, the Pareto chart showed that drying temperature was the most contributing factor in producing the highest total polyphenols content from lemon peel. In contrast, extraction time was not significant as it fell below t-value limit.

**Table 3:** P-value of factors on responses.

Factors	p-values for the responses		
	Y_1_	Y_2_	Y_3_
A	<0.0001	<0.0001	<0.0001
B	0.0007	0.9758	0.0227
C	<0.0001	0.0097	<0.0001
D	0.9051	0.0567	0.3489
E	0.0001	<0.0001	0.0007
Model	<0.0001	<0.0001	<0.0001
Adjusted *R^2^*	0.8396	0.8273	0.8988
C.V. (%)	7.72	11.70	9.09

A – Drying temperature (°C); B – Methanol concentration (%); C – Extraction temperature (°C); D – Extraction time (min); E – Storage duration (day); Y_1_ – Total polyphenols content (mg GAE/g DW); Y_2_ – Total phenolic acids content (mg GAE/g DW); Y_3_ – Total flavonoids content (mg RE/g DW).

**Table 4:** Percentage contribution of factors on responses.

Factors	Percentage contribution (%)		
	Y_1_	Y_2_	Y_3_
A	34.91	38.39	43.55
B	7.17	4.67 × 10^-4^	2.15
C	20.47	3.96	33.27
D	5.91 × 10^-3^	2.01	0.33
E	9.94	14.37	5.37
AB	0.88	2.33	1.03
AC	6.77	1.18	0.31
AD	0.09	1.42	0.05
AE	0.11	19.08	0.02
BC	6.50	0.96	5.16
BD	0.32	0.91	0.30
BE	1.78	0.61	1.4 × 10^-4^
CD	0.17	0.17	0.26
CE	0.21	0.74	1.13
DE	0.50	1.22	1.15

A – Drying temperature (°C); B – Methanol concentration (%); C – Extraction temperature (°C); D – Extraction time (min); E – Storage duration (day); Y_1_ – Total polyphenols content (mg GAE/g DW); Y_2_ – Total phenolic acids content (mg GAE/g DW); Y_3_ – Total flavonoids content (mg RE/g DW).

### 3.2 Influence of extraction factors on the total phenolic acids content

**Fig. 2:**
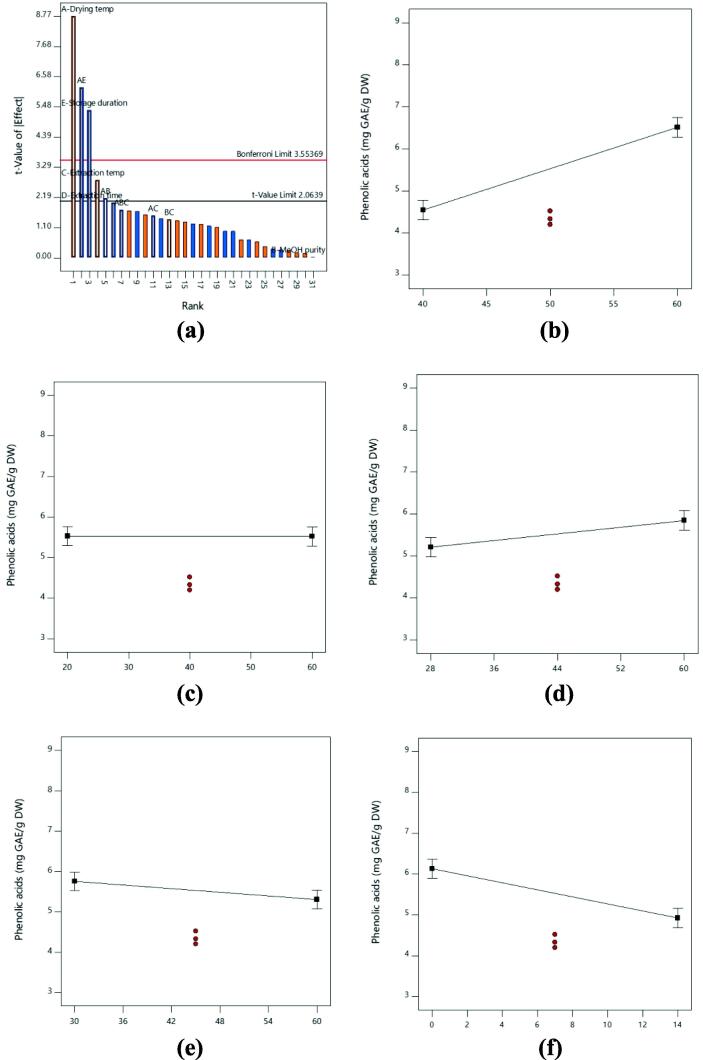
**(a)** The Pareto Chart of all factors and interactions on total phenolic acids content from lemon peel; Influence of **(b)** drying temperature; **(c)** methanol concentration; **(d)** extraction temperature; **(e)** extraction time; **(f)** storage duration on the total phenolic acids content recovery from lemon peel. A greater slope shows a greater influence on the recovery.

For the total phenolics content, the response model was highly significant with P < 0.0001 (Table 3). Based on the Fig. 2, as the drying temperature and extraction temperature increased, the recoveries of total phenolic acids content were increased. For the storage duration, increased in incubation time resulted in lower recovery while methanol concentration and extraction time showed the least effect on total phenolic acids content of lemon peel (Table 3, Fig. 2). The recovery of total phenolic acids content was greatly affected by drying temperature, extraction temperature and storage duration with P < 0.05. Meanwhile, the factors of methanol concentration and extraction time were showed non-significant effect on total phenolic acids content. Based on Table 4, the major contributing factor of total phenolic acids content was drying temperature with 38.39%, followed by storage duration (14.37%) and extraction temperature (3.96%), respectively. Based on Pareto chart (Fig. 2a), drying temperature was the most contributing factor in extracting higher phenolic acids from lemon peel. Meanwhile, methanol concentration was non-significant as it fell below t-value limit (2.0639).

### 3.3 Influence of extraction factors on the total flavonoids content

**Fig. 3:**
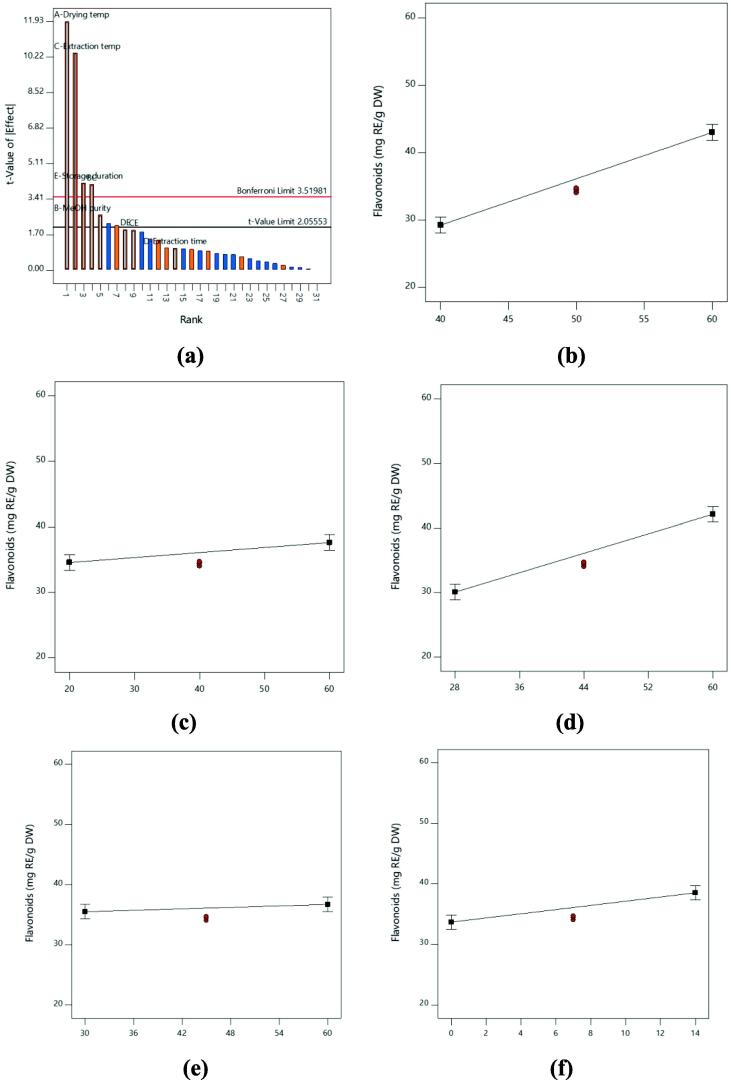
**(a)** The Pareto Chart of all factors and interactions on total flavonoids content from lemon peel; Influence of **(b)** drying temperature; **(c)** methanol concentration; **(d)** extraction temperature; **(e)** extraction time; **(f)** storage duration on the total flavonoids content recovery from lemon peel. A greater slope shows a greater influence on the recovery.

In the analysis of total flavonoids content of lemon peel, the response model was highly significant with P < 0.0001 (Table 3). Based on the Table 3 and Fig. 3, the recovery of total flavonoids content was increased as the drying temperature, methanol concentration, extraction temperature and storage duration were increased. Meanwhile, extraction time exhibited the lowest effect on total flavonoids content. The recovery for total flavonoids content of lemon peel was highly affected by drying temperature, methanol concentration, extraction temperature and storage duration with P < 0.05. Besides that, non-significant effect was shown by extraction time on total flavonoids content. Based on Table 4, drying temperature was the major contributing factor with 43.55%, followed by extraction temperature (33.27%), storage duration (5.37%) and methanol concentration (2.14%), respectively. Based on Pareto chart in Fig. 3a, drying temperature was the highest contributing factor in attaining highest total flavonoids content from lemon peel. Besides that, extraction time was not significantly affected the total flavonoids content as it fell below t-value limit (2.05553).

## 4 Discussion

Among all the factors, drying temperature has the greatest influence on total polyphenols, phenolic acids and flavonoids content of lemon peel. From Table 3, Table 4, this factors showed a highly significant value with P < 0.0001 and it showed the highest percentage contribution for all the responses. Similarly, by comparing the line plots shown in Fig. 1b, 2b and 3b, the effect of drying temperature was clearly shown the greatest gradient of the slope. The total polyphenols, phenolic acids and flavonoids content extracted from the lemon peel was significantly increased as the drying temperature increased from 40 to 60 °C. The trend in our study is similar to previously published results *Solanum lycopersicum* (Gümüşay et al., 2015, Azeez et al., 2019), leafy vegetables (Nwozo et al., 2015) and red rice (Hu et al., 2017) which observed the phenolics content was increased as the drying temperature increased. The accumulation of high phenolics during high drying temperature is related to the increment of the release phenolics bound from the cell wall as a result of ester breaks down between phenolic and cell wall due to the heat treatment (Azeez et al., 2019). As a result, more phenolics would be extracted from the sample.

Extraction temperature also plays an important role in extracting high total polyphenols, phenolic acids and flavonoids content. This factor exhibited highly significant effect on total polyphenols and flavonoids content with P < 0.0001 and significant effect on total phenolic acids content with P < 0.05, respectively (Table 3). Based on Table 4, this factor also showed high percentage of contribution on total polyphenols, phenolic acids and flavonoids content and Fig. 1d, 2d and 3d showed large gradient of the slope. In this study, increment of extraction temperature was significantly increased the total polyphenols, phenolic acids and flavonoids content of lemon peel. The possibilities suggested for the increment of phenolics content might be due to release of high amounts of phenolics due to the thermal destruction of cell walls and sub-cellular compartments. The thermal treatments during extraction process will soften the plant biomass which resulted in higher bioavailability of polyphenols content inside the cell wall. Besides that, heat can break the supramolecular structure of the plant biomass which release more phenolics. As a result, phenolic solubility, diffusion rate and mass transfer are increased while solvent viscosity and surface tension are decreased (Bunea et al., 2008, Nwozo et al., 2015, Mokrani and Madani, 2016). The effect of extraction temperature on phenolic content also have been reported by several previous studies on different types of plant sample (Thoo et al., 2010, Deng et al., 2017, Zhou et al., 2018).

It was also noticeable that storage duration was important in attaining high total polyphenols, phenolic acids and flavonoids content from lemon peel. From Table 3, storage duration was highly significant on total phenolic acids content with P < 0.0001 and significant P-value was obtained on total polyphenols and flavonoids content with P < 0.05. From the line plot in Fig. 2f, negative slope gradient was obtained on total phenolic acids content, contrary with total polyphenols and flavonoids content which were produced the positive slope gradient (Fig. 1f and 3f). This showed that the phenolic acids content was decreased as the storage duration increased. However, the total polyphenols and flavonoids were increased as the storage duration was increased in this study. Polyphenols is one of the secondary metabolites that is known to be unstable during storage especially at ambient temperatures (Howard et al., 2012). However, at low temperature (<10 °C) storage condition, the phenolics and flavonoids in fruits can be retained for several days. The study conducted by Fawole and Opara (2013) on pomegranate found that the total phenolics content was relatively stable for four-week storage duration at a temperature of 5, 7 and 10 °C. The deterioration of total phenolics content was observed at all temperatures after eight-week of storage duration (Fawole and Opara, 2013). The accumulation and deterioration of phenolics during storage as response to storage temperature and duration are mainly affected the genes and enzymes synthesis which is different in each type of food (Galani et al., 2017).

The extraction solvent is one of the factors that highly influenced the extraction of phenolics from plant sample. High polarity of solvent such as aqueous, methanol and ethanol are frequently used as extraction solvent (Dai and Mumper, 2010). In this current study, methanol was chosen to extract the phenolics content from the lemon peel. The uses of methanol as extraction solvent are highly preferred and previously reported this solvent is effective for phenolics extraction (Ye et al., 2015, Vajić et al., 2015). The results in this study found that methanol concentration was significantly affected the total polyphenols and flavonoids content extracted from lemon peel with P < 0.05 (Table 3). However, methanol concentration was not significantly affected the total phenolic acids content with the P-value recorded was more than 0.05. Based on Fig. 1c and 3c, only small gradient slope was obtained on total polyphenols and flavonoids content. In term of percentage of contribution, total phenolic acids content recorded the lowest percentage of contribution than total polyphenols and flavonoids content (Table 4). The total polyphenols and flavonoids content were significantly increased as the methanol concentration increased from 20 to 60%. In contrast with the total polyphenols and flavonoids content, the increment of methanol concentration was not significantly increased the phenolic acids content. In plant, the polyphenols are present at different affinities indicating that different polarities of solvent might directly affecting the phenolics content extraction (Viera et al., 2017). Thus, this study showed that high concentration of solvent produced higher polyphenols and flavonoids content. But, a higher concentration of solvent approximately 80% of solvent is needed to extract higher amount of phenolic acids. According to Chirinos et al. (2007), low phenolics concentration at low concentration of extraction solvent may be due to high amount of water in solvent which is responsible to extract impurities (polysaccharides, organic acids and carbohydrates) may interfere the phenolics extraction.

Extraction time did not show any significant effect on total polyphenols, phenolic acids and flavonoids content in comparison with other factors. The P-value recorded on all responses was more than 0.05 (Table 3). In addition, the percentage contribution recorded also was low (Table 4). In this study, the extraction time was conducted in 30 to 60 min. The non-significant results obtained in all responses might be due to the extraction time was too short. This phenomenon is explained by Fick’s second law of diffusion, predicting that a final equilibrium between the solute concentrations in the solid matrix (plant matrix) and in the bulk solution (solvent) might be reached after a certain time (Silva et al., 2007). Moreover, prolongation of extraction time until reach the maximum limit will increase the chance of sample to denature. Thus, higher phenolics content can be extracted from the sample. In a previous study on *Citrus aurantifolia, Citrus nobilis, Citrus grandis* and *Citrus reticulata*, the optimum extraction time recorded was between 80 and 120 min (Tran et al., 2019, Hien et al., 2018, Colodel et al., 2018, Dao et al., 2020). Hence, it is suggested that the extraction time needed for maximum phenolic extraction from *Citrus limon* is more than 60 min.

## 5 Conclusions

The two-level factorial design was adopted in screening of five independent factors including drying temperature, methanol concentration, extraction temperature, extraction time and storage duration of lemon peel extraction. In attaining high total polyphenols, phenolic acids and flavoanoids content from lemon peel extract, each factor was evaluated to find the most significant with high contribution factor. Results indicated that drying temperature, storage duration and extraction temperature were the most significant and contributing factor affecting the total polyphenols, phenolic acids and flavonoids content of lemon peel with P-value<0.05. Meanwhile, extraction time exhibited the least significance and contribution with P>0.05. Therefore, optimization using the obtained significant factors will be conducted using response surface methodology in the future.

## CRediT authorship contribution statement

**Zainol Haida:** Conceptualization, Methodology, Investigation, Data curation, Software, Formal analysis, Writing - original draft and editing the manuscript. **Sharin Ab Ghani:** Conceptualization, Methodology, Software, Formal analysis, Writing – review and editing. **Jaafar Juju Nakasha:** Project administration, Supervision, Funding acquisition, Resources, Writing - review and editing. **Mansor Hakiman:** Project administration, Supervision, Funding acquisition, Resources, Writing - review and editing. All authors have made direct contribution and this manuscript was approved by all authors for publication.

## Declaration of Competing Interest

The authors declare that they have no known competing financial interests or personal relationships that could have appeared to influence the work reported in this paper.
